# Reduced Graphene Oxide/Organic Dye Composites for Bioelectroconversion of Saccharides: Application for Detection of Saccharides and α-Amylase Assessments

**DOI:** 10.3390/bios13121020

**Published:** 2023-12-08

**Authors:** Marius Butkevicius, Justina Gaidukevic, Vidute Gureviciene, Julija Razumiene

**Affiliations:** 1 Department of Bioanalysis, Institute of Biochemistry, Life Sciences Centre, Vilnius University, Sauletekio Av. 7, LT-10257 Vilnius, Lithuania; marius.butkevicius@gmc.vu.lt (M.B.); vidute.gureviciene@gmc.vu.lt (V.G.); 2 Institute of Chemistry, Faculty of Chemistry and Geosciences, Vilnius University, Naugarduko Str. 24, LT-03225 Vilnius, Lithuania; justina.gaidukevic@chf.vu.lt

**Keywords:** biosensor, reduced graphene oxide, PQQ-dependent glucose dehydrogenase, bioelectrocatalysis, α-amylase, saccharides

## Abstract

In this study, PQQ-dependent glucose dehydrogenase (PQQ-GDH) was immobilized onto reduced graphene oxide (rGO) modified with organic dyes from three different classes (acridine, arylmethane, and diazo); namely, neutral red (NR), malachite green (MG), and congo red (CR) formed three types of biosensors. All three rGO/organic dye composites were characterized by scanning electron microscopy, X-ray photoelectron spectroscopy, and Raman spectroscopy. The impact of three rGO/organic dye modifications employed in bioelectrocatalytic systems on changes in enzyme activity and substrate selectivity was investigated. The highest sensitivity of 39 µA/cm^2^ was obtained for 1 mM of glucose when a rGO_MG/PQQ-GDH biosensor was used. A significant improvement in the electrochemical response of biosensors was attributed to the higher amount of pyrrolic nitrogen groups on the surface of the rGO/organic dye composites. Modifications of rGO by NR and MG not only improved the surfaces for efficient direct electron transfer (DET) but also influenced the enzyme selectivity through proper binding and orientation of the enzyme. The accuracy of the biosensor’s action was confirmed by the spectrophotometric analysis. Perspectives for using the proposed bioelectrocatalytic systems operating on DET principles for total or single monosaccharide and/or disaccharide determination/bioconversion systems or for diagnoses have been presented through examples of bioconversion of D-glucose, D-xylose, and maltose.

## 1. Introduction

The conversion and determination of carbohydrates in various solutions are important in many areas—industry (e.g., bioethanol production from lignocellulose [1,2]), clinical research (e.g., blood glucose, fructose level [3]), and the food industry (e.g., sweetener production [4]). The wide diversity of carbohydrates involved in these areas has led to the development of new schemes of biosynthesis and numerous analytical techniques for monitoring carbohydrate concentrations as well. The methods include chromatography (gas [5], thin-layer [6], and high-performance liquid [7]), capillary electrophoresis [8], and colorimetric or infrared spectroscopy [9]; unfortunately, they do not allow easy and rapid monitoring since they require relatively expensive instruments, advanced analytical skills, and often need a sample pre-treatment step. A promising alternative for new synthesis and analysis methodologies is the bioelectrocatalytic approach because of its sensitivity, straightforward instrumentation, quick detection, and reasonable cost. In progressive systems of this type, it is necessary to ensure the effective transfer of electrons (ET) between the active site of an enzyme and the electrode material. Meanwhile, in that aspect, the compatibility of the electrode material with the enzyme becomes crucial. Usually, to achieve efficacy in bioelectrocatalysis, additional soluble or immobilized ET mediating compounds are used [10]. However, more than 30 years ago, ET was discovered from the enzymatic layer towards the electrode surface without any additional compounds by achieving a direct electron transfer (DET). This field has become particularly promising since DET is a highly desired feature for the development of mediator-free bioelectrocatalytic systems, which, in principle, allow for the simplification of technological performance and cost reduction. Nevertheless, it was demonstrated that the DET phenomenon is restricted by the specificity of the enzyme structure, proper materials for the action of immobilized enzymes, and the distance between the active center of the enzyme and the electrode surface, which should be no longer than 2 nm [11]. Thus, DET crucially depends on the choice of the appropriate immobilization surface [12]. Various nanomaterials and chemical modifications of electrode surfaces are presently being used to optimize the surfaces in order to maximize DET via optimal enzyme binding and orientation. The authors of [13] claim that the bioelectrocatalytic system provides successful DET when the enzyme-modified electrodes employed as biosensors have sensitivities ranging from 31 to 275 nA.

One of the easiest ways to generate a surface for effective enzyme immobilization is by the use of graphene-based nanomaterials like graphene oxide (GO) or reduced graphene oxide (rGO). Graphene is a new kind of two-dimensional single-atom carbon sheet with a single thick atom. It has been extensively used for biosensor research and application. To enhance the electrochemical properties of the GO-based biosensor, GO can be modified with other materials, such as macromolecules, small-mass organic molecules, metallic oxide, and metallic/nonmetallic simple substances [14]. In this research, rGO was modified with different classes of organic dyes and demonstrated how electrode material properties influenced ET in designed bioelectrocatalytic systems. Organic dyes were used for the covalent modification of graphene oxide, which provided different surface functional groups. It is known that some organic dyes enhance the redox capability of GO. Therefore, MG, CR, and NR were selected as they are redox dyes with high nitrogen content, and, to the best of our knowledge, there are no available literature data on the introduction of these dyes in the hydrothermal-assisted modification of GO.

The enzyme PQQ-dependent glucose dehydrogenase from *Acinetobacter calcoaceticus* (PQQ-GDH) is one of those that has been demonstrated to be capable of DET [15]. There are two forms of PQQ-GDH enzymes—soluble (PQQ-sGDH) and membrane bonds (PQQ-mGDH) [16]. PQQ-sGDH demonstrates ability to convert a wider variety of saccharides in a range of monosaccharides such as glucose, galactose, arabinose, and disaccharides such as lactose, cellobiose, and maltose, but not pentoses [16,17], while PQQ-mGDH converts glucose, mannose, galactose, fucose, and xylose, and it does not convert lactose or show a significant reaction with other disaccharides [16]. In this study, PQQ-sGDH was chosen to create the bioelectrocatalytic conversion/detection systems for saccharides (monosaccharides and disaccharides). Using the low specificity of the enzyme, a biosensor was developed that can determine the concentration of saccharides with reducing end in terms of equivalents of a selected saccharide, for example, D-glucose. Also, the biosensor is suitable for use in bioconversion systems when a specific saccharide is converted to the desired product, for example, D-glucose to glucono-δ-lactone. In this work, we are the first to demonstrate that the non-specificity of PQQ-GDH can be used to determine α-amylase activity. Previously, at least two enzymes (α-glucosidase and D-glucose oxidase) were used in biosensors for the determination of α-amylase activity. With a multi-enzyme system, the analysis time is longer than our proposed systems. Some examples of PQQ-GDH biosensors and their parameters are listed in supplementary information Table S7.

PQQ-sGDH was immobilized onto rGO modified with organic dyes from three different classes (acridine, arylmethane, and diazo); namely, neutral red (NR), malachite green (MG), and congo red (CR) formed three types of biosensors. Aiming to understand the effect of modifications used in bioelectrocatalytic systems on DET efficacy and substrate selectivity, all three rGO/organic dye composites were characterized by scanning electron microscopy, X-ray photoelectron spectroscopy, and Raman spectroscopy. The potential applications of proposed DET bioelectrocatalytic systems for total or single monosaccharide and/or disaccharide determination/bioconversion systems, as well as diagnostics, have been discussed. Furthermore, the practical application of the biosensor was exemplified through its utilization in assessing the activity of α-amylase. The determination of α-amylase activity relies on the process of oxidizing saccharides that are produced during the enzymatic reaction. Specifically, an α-amylase catalyzed reaction leads to the formation of maltose and other short dextrins.

## 2. Materials and Methods

### 2.1. Materials

The soluble PQQ-dependent glucose dehydrogenase (EC 1.1. 99.17, 1000 U/mg) was obtained from Creative enzyme. PQQ-GDH activity was measured spectrophotometrically using phenazine methosulfate (PMS) and 2,6-dichlorophenol indophenol (DCPIP) as electron acceptors in 50 mM Tris with 100 mM KCl and 5 mM CaCl_2_, pH 7.2 as described in [18]. (Hydroxymethyl)ferrocene (HMF) purchased from Sigma-Aldrich. All other reagents were used without further purification. Extra pure fine graphite powder (<50 μm, ≥99.5%) was purchased from Merck KGaA (Darmstadt, Germany), HCl (37%) from Carl Roth (Karlsruhe, Germany). H_2_SO_4_ (98%), NaNO_3_ (≥99%), KMnO_4_ (≥99%), H_2_O_2_ (37%), congo red (>90%), neutral red (>90%), malachite green (>90%)—from Sigma-Aldrich (Darmstadt, Germany). 

### 2.2. Synthesis of Graphene Oxide

The synthesis method follows the original procedure developed by Hummers and Offeman [19]. Initially, 6 g of graphite powder was introduced into a cold mixture, maintained at 3 °C, which consisted of 240.0 mL of concentrated H_2_SO_4_ and 3.0 g of NaNO_3_. Then, addition of 30.0 g of KMnO_4_ was carefully performed with strict temperature control to ensure it remained below 10 °C. Then, after stirring for 1 h at 35 °C, the mixture was carefully diluted with 276.0 mL of H_2_O. Subsequently, the mixture was agitated at 70 °C for 15 min, followed by the addition of an extra 840.0 mL of H_2_O. To complete the process, 20.0 mL of 30% H_2_O_2_ was introduced into the mixture. The resulting yellow mixture underwent filtration and was rinsed with a 10 wt% HCl solution (1.0 L) to eliminate any metal ions. The obtained powder then underwent dialysis until the dialysate was free of sulfate and reached a pH of 6. 

### 2.3. Modification of Graphene Oxide with Organic Dyes

**Figure 1:**
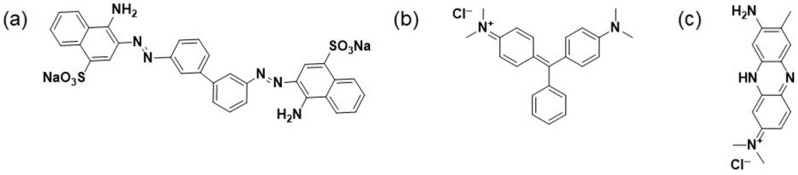
Chemical structures of organic dyes. (**a**) congo red, (**b**) malachite green, (**c**) neutral red.

Composite materials were produced by introducing three different dyes: neutral red, malachite green, and congo red, representing different chemical classes (acridine, arylmethane, and diazo, respectively). The chemical structures of these dyes are illustrated in Figure 1. Three different samples, each with a dye concentration of 20 wt%, were prepared. In each case, 0.1 g of GO was mixed with 0.125 g of the corresponding dye in 50.0 mL of distilled water and agitated for 3 h using a KS 130 Basic Orbital shaker (IKA, Staufen, Germany). Then, the GO/dye suspension underwent 1 h of sonication with a VibraCell VCX-130 (Sonics Inc., Newtown, CT, USA) ultrasonic processor. The resulting GO/dye suspension was transferred to a Teflon-lined autoclave and subjected to hydrothermal treatment at 180 °C for 12 h in a muffle oven. Afterward, the samples were filtered, thoroughly rinsed with deionized water, and dried at room temperature. The materials obtained were designated as rGO_NR, rGO_MG, and rGO_CR, respectively. As a reference, hydrothermal treatment of GO without dye additives was performed, resulting in the sample being identified as rGO. 

### 2.4. Characterization of Graphene Oxide and His Derivation 

The morphology of the samples was studied using the Hitachi SU-70 scanning electron microscope (SEM) (Tokyo, Japan). The applied magnification was 25,000, and accelerating voltage was 10.0 kV.

Raman spectra were obtained using an inVia Raman spectrometer (Renishaw, Gloucestershire, UK) equipped with a thermoelectric cooling CCD camera (70 °C). The He-Ne gas laser supplied with a 532 nm excitation beam with a power limit of 1 mW was used for the measurement. The integration time was 100 s.

Analysis of X-ray photoelectron spectroscopy (XPS) was performed in a Kratos Axis Supra spectrometer (Kratos Analytical, Manchester, UK) using monochromatic Al K radiation (1486.69 eV). Binding energies were standardized in respect to the base C1s peak, which is 284.6 eV. The XPS spectra were deconvoluted by curve-fitting peak components with the CASAXPS software (version 2.3.15) without any pre-smoothing. Following Shirley-type background subtraction, the fitting components’ line shapes were approximated using symmetric Gaussian–Lorentzian product functions. 

### 2.5. Biosensor Fabrication

Before use, the graphite electrode was cleaned with sandpaper and washed several times with acetone and deionized water. For the biosensor fabrication, a mixture was prepared by adding 2 μL of BSA (2.5%), 10 μL of glutaraldehyde (2.5%), 2 μL of PQQ-GDH (1015 U/mL), and 3 μL of pristine GO, either hydrothermally treated GO without dye additives (rGO) or its derivatives aqueous solution (10 mg/mL). rGO or its derivatives solution was made by dissolving 10 mg of solid powder into 1 mL deionized water and sonicating it for 3 h. All mixture was dropped onto a graphite electrode and then air dried (about 1 h). Finally, the pretreated surface of the electrode was covered by the flexible terylene film that was fastened to a rubber ring by forming the biosensor. Prepared biosensor was stored dry at 4 °C. 

### 2.6. Electrochemical Measurements

All electrochemical measurements were obtained using an electrochemical system (Bioanalizes sistemos, Vilnius, Lithuania) with a conventional three-electrode—an auxiliary electrode (4 mm, platinum disk), a reference electrode (Ag/AgCl 3M), and a working electrode (biosensor designed as described above). All calibration and sample analyzing measurements were performed under potentiostatic conditions at 0.4 V vs. Ag/Ag/Cl 3M in a stirred 50 mM Tris with 100 mM KCl and 5 mM CaCl_2_, pH 7.2, buffer solution, t = 25 °C. The experimental data (dependence of the current density (j) on substrate concentration (S)) were approximated to the electrochemical version of the Michaelis–Menten Equation [20] with substrate inhibition (1).

(1) $$j=\frac{j_{max}*S}{K_{m}^{app}+S*(1+\frac{S}{K_{i}})}$$

where $K_{m}^{app}$ is apparent Michaelis constant, $j_{max}$—maximal current density, $K_{i}$—inhibition constant. The geometric surface area of the working electrode (0.07 cm^2^) was used to calculate the current density.

### 2.7. Sulfuric Acid–UV Method

The concentration of saccharide solutions was determined using sulfuric acid–UV method [21]. Shortly, 0.5 mL aliquot of saccharide solution in water is rapidly mixed with 1.5 mL of concentrated sulfuric acid in a test tube and vortexed for 10 min. The solution was then brought to room temperature by being chilled in ice for 2.5 min. Finally, absorption at 315 nm was recorded using UV spectrophotometer. By following the same procedures as above but using deionized water instead of the saccharide solution, reference solutions were made.

### 2.8. α-Amylase Activity Determination

The electrochemical determination of α-amylase activity consists of two steps. A total of 2 µL of saliva sample was added into measuring cell and filled with 1.7 mL buffer solution (50 mM Tris with 100 mM KCl and 5 mM CaCl_2_, pH 7.2), then 150 µL of 1.5% starch solution was added. Addition of starch and the presence of amylase in the solution initiated a hydrolysis reaction to form maltose and other short maltodextrins. Maltose acts as a substrate for PQQ-GDH, and an increase in current is observed during the re-agentless bioelectrochemical reaction. The rate (current change in time) of maltose oxidation is directly proportional to the concentration of α-amylase. Calibration with a solution of α-amylase of known concentration is carried out before testing.

The colorimetric determination of α-amylase using the commercial spectrophotometer kit (Amylase Activity Assay Kit, Sigma-Aldrich, Darmstadt, Germany) was performed as instructed by the guideline provided with the kit.

## 3. Results

### 3.1. Characterization of rGO and rGO/Organic Dye Composites

**Figure 2:**
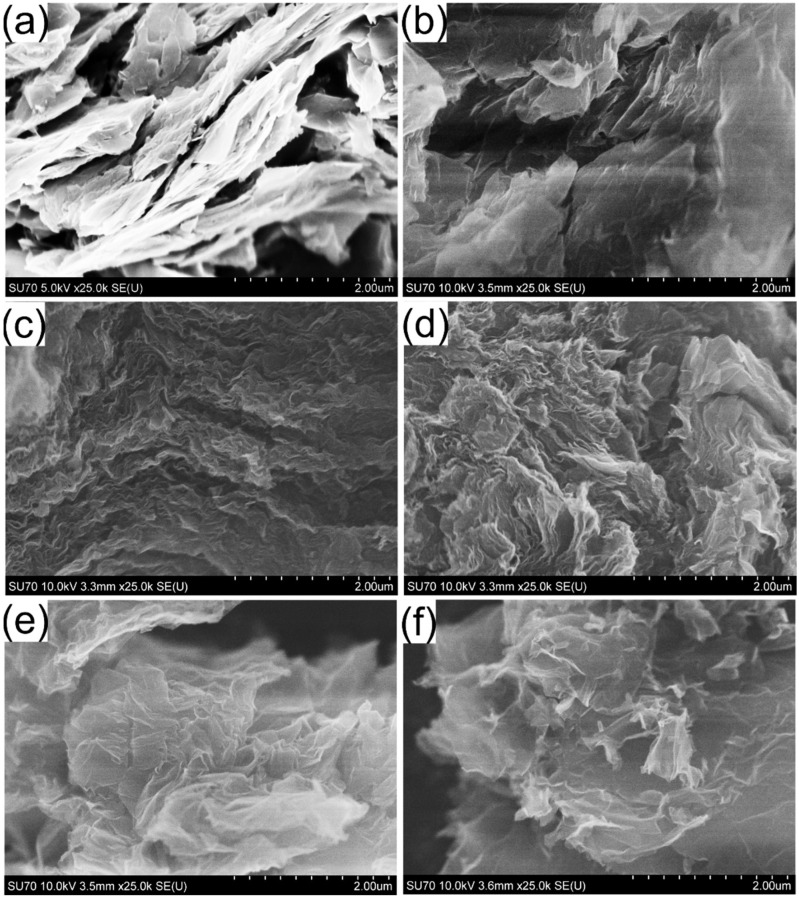
SEM images of graphene-based samples. (**a**) graphite, (**b**) GO, (**c**) rGO, (**d**) rGO_CR, (**e**) rGO_NR, (**f**) rGO_MG.

SEM analysis was used to show the structural evolution of GO before and after hydrothermal treatment. The results are presented in Figure 2. As can be seen in Figure 2a, the layered crystalline structure of graphite is clearly observable and is different from the other samples. The structure of GO changes dramatically (Figure 2b). The chemical treatment of graphite with a strong oxidizing agent leads to the expansion of the d-spacing of graphite and a decrease in the number of stacked layers. As a consequence, the corrugated morphology of GO is observed in the SEM images. The rGO structure consists of stacked individual carbon sheets (Figure 2c). Hydrothermal treatment of GO results in obtaining a typical “worm-like” turbostratic structure. Compared to rGO, the hydrothermal treatment of GO in the presence of organic dyes exhibits a similar microstructure. However, the characteristic changes in the morphology of the samples are visible. From Figure 2d–f, it can be noted that crumpled graphene nanosheets are randomly arranged and overlapped with each other. The wrinkles observed at the edge of the graphene nanosheets are most likely due to the incorporation of nitrogen and/or sulfur species into the graphitic network.

**Figure 3:**
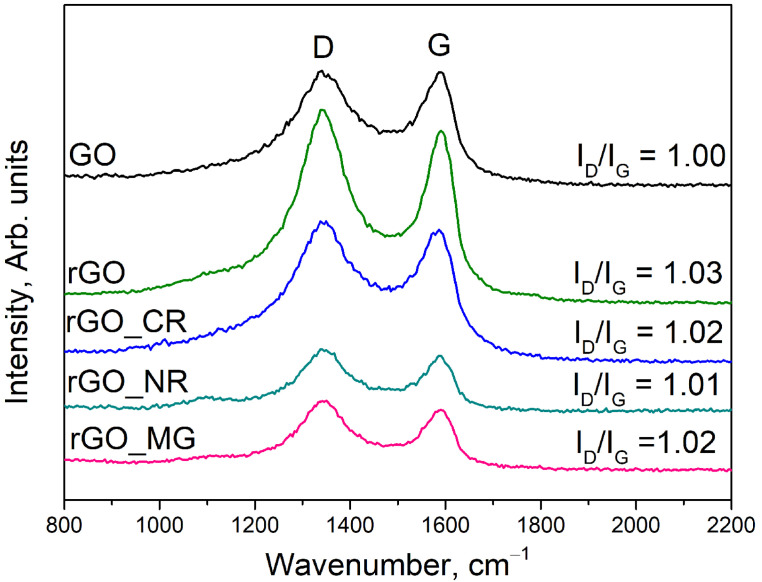
Raman spectra of GO, rGO, and rGO/organic dye composites.

In order to identify the structural changes during the hydrothermal treatment of GO in the absence and presence of organic dyes, the products obtained were investigated by Raman spectroscopy. Raman spectra are depicted in Figure 3. 

The typical Raman spectra of GO are characterized by D and G bands at approximately 1341.56 cm^−1^ and 1586.69 cm^−1^, respectively. According to the literature, the G mode represents the symmetry and sp^2^ hybrid structure of carbonaceous materials, while the D mode demonstrates the defects and imperfections of graphene layers, such as C vacancies, sp^3^ defects, etc. [22,23]. The intensity ratio of the D and G modes is applied to determine the degree of disorder in graphene-based materials. The higher the I_D_/I_G_ ratio, the more lattice distortions are presented in the structure of the graphene layers [24]. 

Compared to graphite (I_D_/I_G_ = 0.10) [25], the I_D_/I_G_ value of GO increases up to 1.00, indicating a considerably higher degree of structural disorder and a higher number of sp^3^-like defects in the graphene layers due to a strong oxidation process. In addition, after hydrothermal treatment of GO, the I_D_/I_G_ ratio of the final product slightly increased from 1.00 to 1.03. As a result, the additional formation of structural defects onto graphene layers could be caused by the removal of oxygen-containing functional groups present on the surface of GO. Furthermore, a similar trend of increase in the I_D_/I_G_ values is also observed in the case of the hydrothermal treatment of GO in the presence of organic dyes. One reasonable explanation for this increase could be that hydrothermal treatment of GO with organic dye additives causes the removal of oxygen functionalities and the introduction of N and/or S atoms into the graphene lattice. The incorporation of dopants disordered the two-dimensional hexagonal lattices of graphene, and thus more surface defects occur in the rGO/organic dye composites than GO.

**Figure 4:**
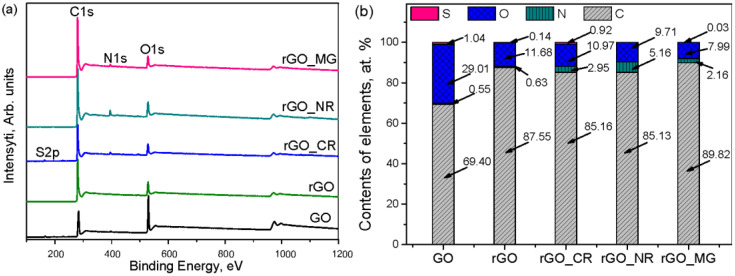
The survey XPS spectra (**a**) and surface elemental composition (**b**) of GO, rGO, and rGO/organic dye composite samples determined by the XPS.

The chemical analysis of the prepared graphene-based materials was carried out by XPS (Figure 4). Figure 4a,b show the wide scan XPS spectra and the elemental compositions of all prepared samples, respectively. From wide scan XPS spectra, it can be noted that elements including C, O, N, and S were presented in the GO and rGO samples. Moreover, as can be seen, the oxidation of graphite led to significant incorporation of oxygen-containing functional groups, whereas hydrothermal treatment of the resulting GO was connected with a large reduction in the oxygen content (from 29.01 at.% to 11.68 at.%). Also, it was found that a small amount of sulfur and nitrogen was attached to the surface of GO (S 1.04 at.%; N 0.63 at.%) and rGO (S 0.14 at.%; N 0.55 at.%). These elements may be derived from the pristine sulfur- and nitrogen-containing reagents used in the synthesis. Further, the XPS data revealed that after the hydrothermal treatment of GO in the presence of organic dyes, the N-containing functional groups were successfully attached to the carbon framework. The concentration of nitrogen increased from 0.63 at.% in GO to 2.16–5.16 at.% in the modified samples. Thus, the highest nitrogen content was shown by the rGO_NR sample (5.16 at.%) and the lowest for the rGO_MG sample (2.16 at.%).

**Figure 5:**
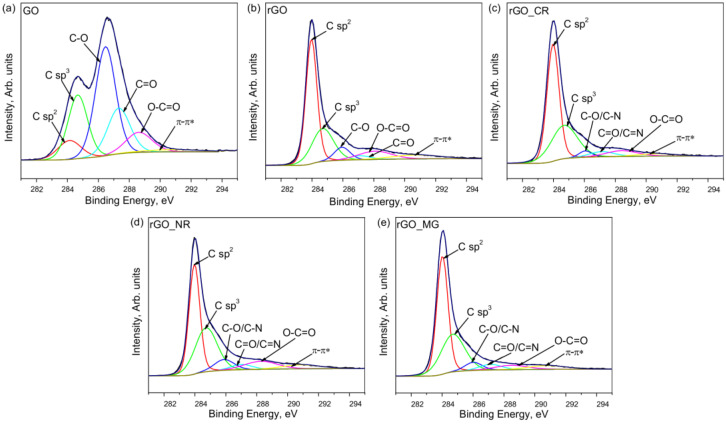
XPS C1s spectra of prepared graphene-based samples: GO (**a**), rGO (**b**), rGO_CR (**c**), rGO_NR (**d**), and rGO_MG (**e**).

Figure 5 displays the C1s peaks of the GO, rGO, and rGO/organic dye composites. The C1s of GO consist of six peaks that are attributed to the sp^2^ C and sp^3^ C in the aromatic rings, oxygen-containing functional groups such as hydroxyl/epoxy, carbonyl, and carboxyl, and π–π* graphitic shake-up satellites. These assignments agree with previous work [26,27]. The high-resolution C1s XPS spectrum of rGO (Figure 5b), while showing the same oxygenated functional groups as GO, had a smaller concentration of these groups (Table S1), indicating partial de-oxygenation by the hydrothermal treatment. Also, after hydrothermal treatment in the presence of organic dyes, the peak at ~285.94 eV (for rGO) is slightly shifted to 286.06 eV (for rGO_CR), 286.02 eV (for rGO_MG), and 286.06 (for rGO_NR). Similarly, the peak at ~287.05 eV (for rGO) moved to 287.13, 287.16, and 287.20 eV in the case of rGO_CR, rGO_MG, and rGO_NR, respectively. Moreover, in comparison to the rGO sample, the concentration of the C sp^3^ and C=O bands in the case of the rGO/organic dye composite was slightly increased (Table S1). Both of these facts suggest the attachment of N-containing groups in the structure of the samples.

**Figure 6:**
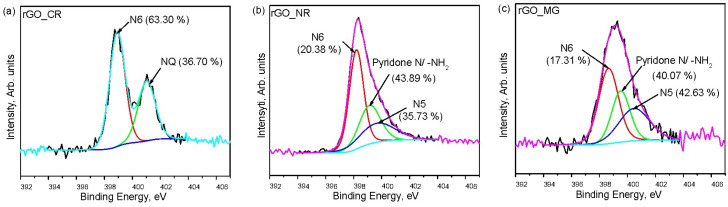
XPS N1s spectra of rGO/organic dye composite samples: rGO_CR (**a**), rGO_NR (**b**), and rGO_MG (**c**).

Further, to better characterize the N-containing functional groups formed during the hydrothermal treatment of GO in the presence of organic dyes, the deconvolution of the N1s spectra has been performed. The results depicted in Figure 6. verify the presence of various nitrogen functionalities. The high-resolution N1s spectra of the rGO_CR sample can be deconvoluted into two main peaks: at the binding energies of 398.92 eV and 401.03 eV, corresponding to pyridinic nitrogen (N6) and quaternary nitrogen (NQ), respectively [28,29]. On the contrary, the deconvolution of the N1s spectrum of rGO_NR and rGO_MG shows three signals. The peaks centered at approximately 398.66 eV (for rGO_MG) and 398.20 eV (for rGO_NR) can be assigned to the pyridinic nitrogen (N6). Further signal at about 399.50 eV (for rGO_MG) and 399.14 eV (for rGO_NR) belongs to pyridone (O=C-N-C) or amine (-NH2) functionalities. The remaining peak with binding energies of approximately 400.48 eV (for rGO_MG) and 400.00 eV (for rGO_NR) correspond to pyrrolic nitrogen (N5) [29,30]. Also, based on the results presented in Figure 6, it can be noted that the pyridinic nitrogen (63.30 at.%) is the most abundant on the surface of rGO_CR. Also, a smaller amount of N5 (36.70 at.%) group is present on its surface. Instead, the pyridone N/amine (43.89 at.%) groups were dominated onto the rGO_NR surface. In the case of the rGO_MG sample, a higher amount of pyrrolic nitrogen groups are attached to the surface. However, compared to the hydrothermal treatment in the presence of congo red dye, treatment with NR and MG additives causes a considerable decrease in the pyridinic N (20.38 at.% for rGO_NR; 17.31 at.% for rGO_MG) groups and the absence of quaternary nitrogen was observed.

### 3.2. Bioelectrocatalytic Properties of rGO and rGO/Organic-Dye-Composite-Based Biosensors

**Figure 7:**
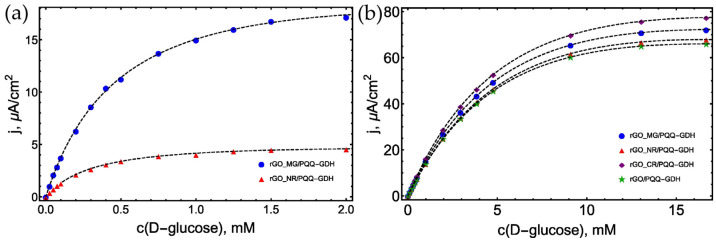
(**a**) Calibration curves for saccharides biosensors. (**b**) Calibration curves for saccharides biosensors with additional soluble mediator (HMF 0.25 mM). A total of 50 mM Tris buffer solution with 100 mM KCl and 5 mM CaCl_2_, pH 7.2, an applied potential of 0.4 V vs Ag/AgCl, t = 25 °C.

Aiming to test the ability of PQQ-GDH to catalyze the oxidation of D-glucose and other sugars, chronoamperometric measurements were performed with four biosensor versions—rGO/PQQ-GDH (control), rGO_NR/PQQ-GDH, rGO_CR/PQQ-GDH, and rGO_MG/PQQ-GDH. First, the response of the manufactured biosensors to D-glucose was recorded as a difference between the steady-state current and the background current. Control rGO/PQQ-GDH and rGO_CR/PQQ-GDH biosensors do not show any bioelectrochemical response to D-glucose and maltose (Figure S1 in the Supplementary Material). Conversely, the biosensors based on rGO_NR/PQQ-GDH and rGO_MG/PQQ-GDH were active toward the bioelectrooxidation of the target analytes. Figure 7a shows the dependences of steady-state current densities on D-glucose, reflecting the effectiveness of DET of used biosensors. To verify that in all versions of biosensors, the enzyme is indeed immobilized and remains active, an additional measurement was performed with the addition of 0.25 mM of the soluble mediator (hidroxymethyl)ferrocene (HMF) into the electrochemical cell. Calibration curves obtained with HMF showed that the activity of PQQ-GDH was achieved in all cases, and the sensitivity of the biosensors was very similar, even in a case using a control biosensor (Figure 7b). Sensitivity to 1 mM of glucose varied from 12.51, 14.56, 13.58, to 12.69 µA/cm^2^ for rGO/PQQ-GDH, rGO_CR/PQQ-GDH, rGO_MG/PQQ-GDH, and rGO_NR/PQQ-GDH, respectively. Apparent Michaelis constant ($K_{m}^{app}$) calculated for these biosensing systems also remained almost the same in the range of 8.52, 7.92, 8.33, and 8.83 mM for rGO/PQQ-GDH, rGO_CR/PQQ-GDH, rGO_MG/PQQ-GDH, and rGO_NR/PQQ-GDH, respectively. Similarities in the sensitivity and linear ranges of biosensors indicate that the kinetics of these biosensors are determined not by DET but by the action of a soluble mediator HMF added into the electrochemical cell. Confirmation of this is a completely different picture was obtained with biosensors using only rGO/organic dye composites (Table S2). 

More than 10 times less linear ranges, and it reflecting $K_{m}^{app}$ values of 0.33 and 0.55 mM for rGO_NR/PQQ-GDH and rGO_MG/PQQ-GDH (Figure 7a), indicating the biosensor action ascertained by the unique characteristics of the composites used. In contrast to the completely inactive rGO_CR/PQQ-GDH, surprisingly high sensitivity to 1 mM of glucose (39 µA/cm^2^) was obtained with the rGO_MG/PQQ-GDH biosensor. Thus, using rGO_MG composite for the design of rGO_MG/PQQ-GDH allowed an increase in the sensitivity of biosensor up to three times compared to bioelectrocatalysis in the presence of soluble ET mediator HMF (Figure 7b). 

To check whether the efficacy of the biosensor is affected either by the surface functional group or organic dyes themselves can act as different ET rate-possessing mediators, an experiment using rGO/PQQ-GDH and adding free organic dye (CR, MG, or NR) as mediators to the solution was performed. According to Figure S2, it appears that organic dye can serve as a mediator and that different dyes have varying degrees of efficacy. However, ET efficacy was rather low since the sensitivity of the biosensor may be increased up to 18 times by employing rGO MG as a composite as compared to a scenario where only dye MG was introduced to the cell where the rGO/PQQ-GDH biosensor functioned. (Figure S2). 

Thus, these results suggest that modification of the sample and functionalities presented on the structure of the sample were critical for the ET in the bioelectrochemical system. No correlation was obtained in the effectiveness of ET, which was expressed as sensitivity and the total nitrogen content (at.%), since a relatively poor electrocatalytic performance was observed for the rGO_NR, which possesses the highest nitrogen content (5.16 at.%) (Figure 4b). The rGO_MG biosensor showed the highest response, while its total nitrogen was 2.16 at.%. A significant improvement in the electrochemical response at the rGO_MG biosensor could be attributed to the higher amount of pyrrolic nitrogen (N5) groups because it was found to have the highest amount of N5 (42.63%) on its surface. In the composite rGO_NR, the N5 amount was 35.73%, and no amount was found in the composite rGO_CR (Figure 6). These findings confirm those of earlier studies, which have demonstrated that carbon-based materials with a relatively high content of pyrrolic nitrogen at the edges of graphene layers will show better charge mobility and donor-acceptor properties compared to pyridinic and graphitic nitrogen [31,32]. This implied that the electrocatalytic activity was influenced more by the presence of pyrrolic nitrogen than by the total nitrogen content. These results are in line with those obtained by other studies, which have shown that nitrogen species and not just the nitrogen content in graphene-based materials play a significant role in influencing the electrocatalytic properties [32,33].

### 3.3. Selectivity of rGO_MG and rGO_NR Based Biosensors

It has already been shown in previous works that the immobilization process of PQQ-dependent enzymes leads to a change in the substrate specificity of the native enzymes [34]. This substrate specificity can be caused by a distortion of the enzyme active center during the immobilization procedure. To immobilize the enzyme, we use the crosslinking method, which can affect the structure of the enzyme by interacting with its amino acids, especially lysine. Based on literature data, mutagenesis studies have shown that the substitution of Lys166 can influence the specificity of the enzyme, especially for D-galactose [35]. Since, in our case, there is a very large change in the specificity of D-galactose, we assume that the glutaraldehyde used during the immobilization of the enzyme interacts with the lysine amino group and a change in the 3D structure of the enzyme occurs, which causes changes in the specificity and structure of the enzyme. Indeed, another factor affecting the change in biosensor specificity is the influence of carbon structures on the path of electron transport. This is confirmed by the different values of $K_{m}^{app}$ determined using the same enzyme and two carbonaceous materials possessing different modifications (rGO_NR, rGO_MG) for a set of substrates (Table S2). We suggest that the PQQ-GDH undergoes conformational changes when it binds to the electrode surface during immobilization, producing the precise spatial orientation of the enzyme and appropriate distance required for a DET in a bioelectrocatalytic conversion reaction.

**Figure 8:**
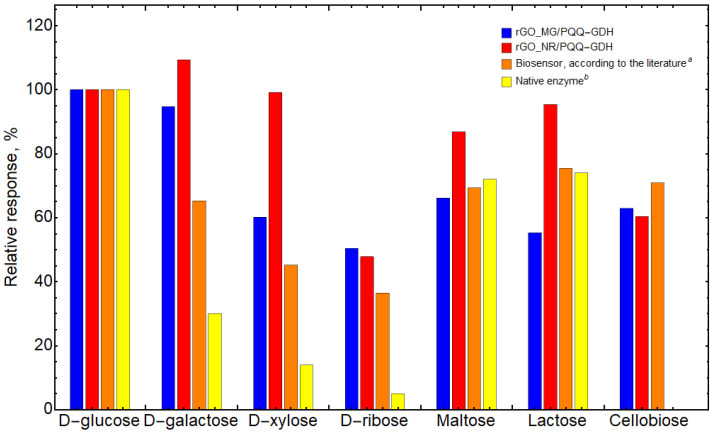
Relative selectivity of biosensors (%). The response to 0.1 mM of glucose was taken as 100%. ^a^ [36], ^b^ [16].

The selectivity of PQQ-GDH immobilized on rGO_MG and rGO_NR was investigated using concentrations of 0.1 mM, which is in the linear range of the D-glucose calibration curve. The response to 0.1 mM of D-glucose was taken as 100%. As seen in Figure 8, in contrast to the rather bad action of the native enzyme, PQQ-GDH catalyzes the oxidation of other substrates, such as cellobiose and ribose, while working on a surface of composites rGO_MG and rGO_NR.

Analysis of $K_{m}^{app}$ values revealed that when the enzyme is operating in the biosensors rGO_MG/PQQ-GDH and rGO_NR/PQQ-GDH, $K_{m}^{app}$ value (Table S2) is decreased by an order compared to the $K_{m}^{app}$ values (ca. 8 mM) obtained in bioelectrocatalysis using a soluble ET mediator. The lower $K_{m}^{app}$ values for rGO_MG/PQQ-GDH and rGO_NR/PQQ-GDH indicate that the pathway by which the substrate’s access to the active center becomes shorter due to the conformation of the enzyme structure during the immobilization.

### 3.4. Stability of rGO_MG and rGO_NR Based Biosensors

**Figure 9:**
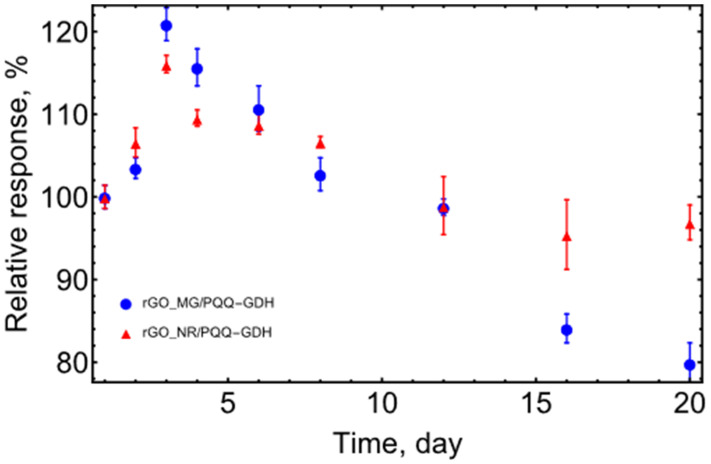
Stability of biosensors. A total of 50 mM Tris buffer solution with 100 mM KCl and 5 mM CaCl_2_, pH 7.2, an applied potential of 0.4 V vs. Ag/AgCl, t = 25 °C.

The stability of the biosensors prepared with PQQ-GDH and GO modified with organic dyes was studied towards D-glucose at room temperature (≈25 °C). For stability evaluation, continuous measurements (at least three measurements per day) of the catalytic current were carried out. Between the measurements, the biosensors were stored at room temperature in a buffer solution. The rGO_MG/PQQ-GDH and rGO_NR/PQQ-GDH electrodes exhibited quite good stability for at least 20 days (Figure 9). An increase in activity is noticeable after 1-3 days, which can be attributed to the swelling of the membrane with an enzyme and rGO [37], as the electrodes after fabrication were kept dry at 4 °C until they were used.

### 3.5. Theoretical Application of Biosensor

**Figure 10:**
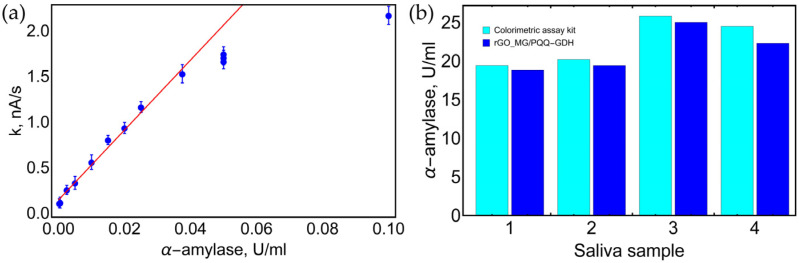
Determination of α-amylase activity in human saliva. (**a**) rGO_MG/PQQ-GDH biosensor calibration curve with different amounts of α-amylase (Red line—linear mathematical model). (**b**) a comparative histogram demonstrating α-amylase activity in human saliva samples measured with rGO_MG/PQQ-GDH and compared with commercial colorimetric assay kit. A total of 50 mM Tris buffer solution with 100 mM KCl and 5 mM CaCl_2_, pH 7.2, t = 25 °C.

Aiming to demonstrate the practical applicability of the proposed biosensors, the rGO_MG/PQQ-GDH biosensor was used to determine (i) monosaccharides (D-glucose and D-xylose) and disaccharides (maltose) in water-based-solutions and (ii) α-amylase activity in human saliva samples. The proposed biosensor has low selectivity for saccharides with reducing ends, so it detects products formed in a reaction catalyzed by α-amylase (e.g., maltose and other short maltodextrins). Before measurements with human saliva, the calibration curve of the rGO_MG/PQQ-GDH biosensor was obtained (Figure 10a) with linearity to α-amylase in a range of 0.005–0.04 U/mL. Using this calibration curve, the oxidation rates of saccharides were recalculated to α-amylase activity. It was found that α-amylase activities (sample dilution was considered) varied in the range of 19.4 to 25.6 U/mL. Additionally, the commercial amylase activity assay kit was used to analyze the saliva samples. A comparison of the results obtained with the rGO_MG/PQQ-GDH biosensor and the commercial kit is presented in Figure 10b. The results demonstrate a good correlation between the rGO_MG/PQQ-GDH biosensor and an alternative colorimetric method since the difference between methods did not exceed 10%.

**Figure 11:**
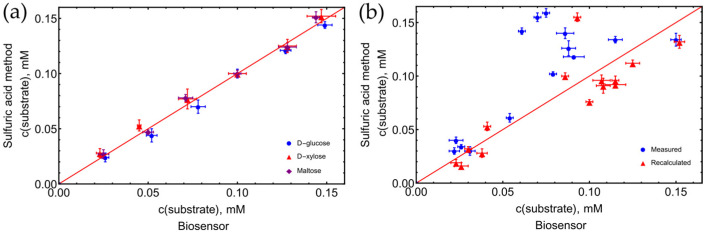
Correlation between results obtained using rGO_MG/PQQ-GDH biosensor and spectrophotometric UV-VIS method. (**a**) Single saccharides solutions. (**b**) Mixture of saccharides. Recalculated values are obtained by considering the specificity of the biosensor and the different absorption of saccharides and H_2_SO_4_ mixture, red line—theoretical linear correlation. A total of 50 mM Tris buffer solution with 100 mM KCl and 5 mM CaCl_2_, pH 7.2, t = 25 °C.

The biosensor rGO_MG/PQQ-GDH was applied for the quantification of monosaccharides (D-glucose and D-xylose) and disaccharides (maltose) as well. The detected analyte concentration was evaluated from the calibration curve based on bioelectrocatalytic responses of known concentrations of the analyte. The same samples of the reaction mixture were also analyzed by the alternative spectrophotometric method (sulfuric acid–UV method [21]. The accuracy of the amperometric biosensor has been confirmed by plotting the results obtained by the amperometric biosensor versus the results obtained using the spectrophotometric analysis (Figure 11a).

The correlation coefficients (R) between the spectrophotometric and rGO_MG/PQQ-GDH biosensor-based analysis were found to be 0.993, 0.989, and 0.991, respectively, for D-glucose, D-xylose, and maltose. These results indicated an excellent agreement between both methods. On this principle, more saccharide mixtures have been tested as well. A larger distribution of values is obtained when analyzing the mixtures and using a 0.1 mM D-glucose response as a standard (Figure 11b). All measured values, mixture composition, and calculated concentration are presented in supporting information Tables S4–S6.

## 4. Conclusions

Four biosensor versions, rGO_NR/PQQ-GDH, rGO_CR/PQQ-GDH, rGO_MG/PQQ-GDH, and rGO/PQQ-GDH (control), constructed using the hydrothermal treatment of GO in the absence and presence of organic dyes demonstrated different sensitivities and even selectivities. Despite the fact that PQQ-GDH has been shown to be capable of DET, it was demonstrated that control, rGO/PQQ-GDH, and rGO_CR/PQQ-GDH biosensors do not show any bioelectrochemical response to D-glucose and maltose without the addition of soluble ET mediator. In contrast to the completely inactive rGO_CR/PQQ-GDH, the highest sensitivity of 39 µA/cm^2^ to 1 mM of glucose was obtained with rGO_MG/PQQ-GDH biosensor operating by DET. This value of sensitivity is up to three times higher compared to that of bioelectrocatalysis in the presence of the soluble ET mediator HMF. After the addition of a soluble mediator, the sensitivity of all biosensors was found to be very similar, even in the case of the control biosensor. $K_{m}^{app}$ calculated for these biosensors also remained almost the same. Similarities in the sensitivity and linear ranges of biosensors indicate that the kinetics of all biosensors were determined by the action of a soluble mediator HMF added to the electrochemical cell. 

The immobilization of PQQ-GDH onto rGO_MG and rGO_NR allowed for a change in the substrate specificity of the native enzyme. Bioelectrocatalytic conversion of ribose and cellobiose was achieved using rGO_MG/PQQ-GDH and rGO_NR/PQQ-GDH, while native PQQ-GDH hardly converts at all these saccharides. The rGO_MG/PQQ-GDH and rGO_NR/PQQ-GDH electrodes exhibited quite good stability for at least 20 days. Compared to previous systems using PQQ-GDH [38,39,40], the proposed system works more efficiently, which opens up new possibilities to apply the biosensor for the quantification of monosaccharides and disaccharides by leading to the development of new schemes of biosynthesis and numerous analytical techniques for monitoring carbohydrate concentrations as well.

## Data Availability

The data presented in this study are available in supplementary material.

## Supplementary Materials

The following supporting information can be downloaded at https://www.mdpi.com/article/10.3390/bios13121020/s1, Figure S1. rGO_CR/PQQ-GDH and rGO/PQQ-GDH current time responses to D-glucose. Figure S2. Calibration curves for rGO/PQQ-GDH electrode obtained using dyes (MG, NR and CR) as additional, soluble mediators. Table S1. Distribution and concentration (at.%) of carbon and oxygen bonds determined by XPS. Table S2. Biosensors characteristics obtained for different saccharides. Table S3. Extinction coefficients for investigated saccharides. Table S4. Theoretical composition of saccharide samples. Table S5. Comparison of theoretical and calculated amounts of D-glucose, Maltose, and D-xylose utilizing amperometric biosensor responses and spectrophotometric analysis. Table S6. Concentrations of saccharides obtained by the amperometric biosensor and using spectrophotometric analysis. Table S7. Comparative performance of bioelectrode based on PQQ-GDH.
