# Supplementary material for: Reduced Graphene Oxide/Organic Dye Composites for Bioelectroconversion of Saccharides: Application for Detection of Saccharides and α-Amylase Assessments
Source: Biosensors (Basel). 2023 Dec 8;13(12):1020. doi: 10.3390/bios13121020 (PMC10741938; doi:10.3390/bios13121020)
Supplement: Supplementary file 1 [file biosensors-13-01020-s001.zip › biosensors-2644912-supplementary.pdf]

Supplementary material

Reduced graphene oxide/organic dyes composites for bioelectroconversion of saccharides: application for detection of saccharides and α-amylase assessments

Marius Butkevicius[1], Justina Gaidukevic[2], Vidute Gureviciene[1], Julija Razumiene[1*]

[1]Department of Bioanalysis, Institute of Biochemistry, Life Sciences Center, Vilnius University, Sauletekio av. 7, LT-10257 Vilnius, Lithuania.

[2]Institute of Chemistry, Faculty of Chemistry and Geosciences, Vilnius University, Naugarduko str. 24, LT-03225 Vilnius, Lithuania.

[*]Corresponding author

Table S1. Distribution and concentration (at. %) of carbon and oxygen bonds determined by XPS.

Sample	C sp^2	C sp^3	C-O/C-N	C=O/C=N	O-C=O	π-π*
GO	13.57	19.71	32.70	21.59	10.50	1.93
rGO	49.34	24.62	7.09	2.97	9.38	6.59
rGO_CR	51.89	31.16	2.87	4.55	6.40	3.16
rGO_NR	41.48	33.3	7.26	3.83	8.96	5.18
rGO_MG	48.43	31.95	5.01	4.47	5.10	5.05

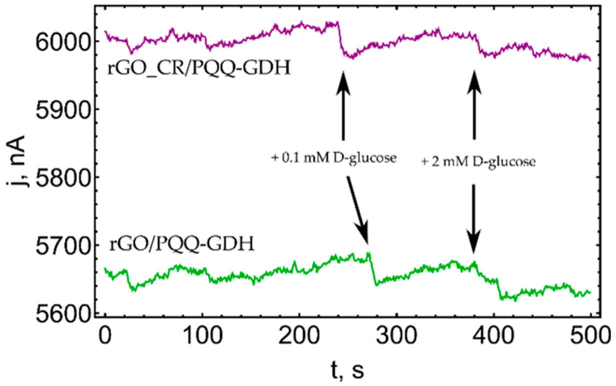

Figure S1. Current time responses to D-glucose. Arrows indicate the addition of saccharide. Measurements were performed under potentiostatic conditions at 0.4 V vs. Ag/AgCl in a stirred 50 mM Tris buffer solution containing 100 mM KCl and 5 mM $CaCl_2$, pH 7.2, and t = 25 ℃. Purple line— rGO_CR/PQQ-GDH; green— rGO/PQQ-GDH electrode.

Table S2. Biosensors characteristics obtained for different saccharides

rGO_NR/PQQ-GDH					
Substrate	K_m^{app}, mM	Vmax, uA/cm^2	K_i	Sensitivity, uA/cm^2*mM	Linear range, mM
D-glucose	0.327	5.71	22.31	14.83	0.025 - 0.1
Maltose	0.407	5.72	16.31	11.25	0.025 - 0.1
D-galactose	0.264	5.17	22.31	17.59	0.025 - 0.1
Lactose	0.308	5.09	6.31	14.17	0.025 - 0.1
D-ribose	0.431	3.48	29.12	6.77	0.025 - 0.1
D-xylose	0.408	6.55	10.30	14.27	0.025 - 0.1
Celobiose	0.578	5.35	10.54	8.31	0.025 - 0.15
rGO_MG/PQQ-GDH					
Substrate	K_m^{app}, mM	Vmax, uA/cm^2	K_i	Sensitivity, uA/cm^2*mM	Linear range, mM
D-glucose	0.547	24.36	16.31	39.01	0.025-0.15
Maltose	0.897	25.86	14.31	20.44	0.025-0.2
D-galactose	0.623	25.79	21.31	35.59	0.025-0.15
Lactose	0.958	22.01	26.31	20.78	0.025-0.2
D-ribose	0.848	17.91	35.31	18.92	0.025-0.2
D-xylose	0.481	13.14	30.31	22.59	0.025-0.15
Celobiose	0.618	16.98	38.31	23.64	0.025-0.15

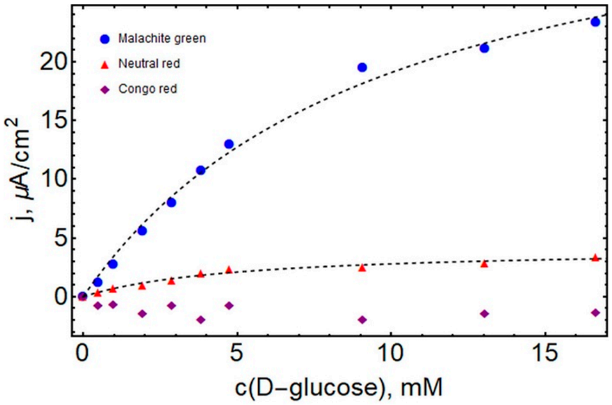

Figure S2. Calibration curves for rGO/PQQ-GDH electrode obtained using dyes (MG, NR and CR) as additional, soluble mediators. Blue dots—0.25 mM MG; red triangles—0.25 mM NR; purple rhombus—0.25 mM CR. A total of 50 mM Tris buffer solution with 100 mM KCl and 5 mM $CaCL_2$, pH 7.2, an applied potential of 0.4 V, t = 25 ^oC.

Table S3. Extinction coefficients for investigated saccharides.

Saccharide	ε, mM^{-1} cm^{-1}
D-glucose	47 ± 2
D-xylose	70 ± 3
Maltose	85 ± 3

D-galactose	48 ± 2
Lactose	69 ± 2
Cellobiose	90 ± 3
D-ribose	43 ± 1

Table S4. Theoretical composition of saccharide samples.

Sample	Composition, %
1	33.3 % D-glucose, 33.3 % Maltose, 33.3 % D-xylose
2	50% D-glucose, 50% Maltose
3	50% D-glucose, 50% D-xylose
4	50 % Maltose, 50% D-xylose
5	66.7 % D-glucose, 33.3 % D-xylose
6	80% D-glucose, 20% Maltose
7	25% D-glucose, 25% Maltose, 25 % D-xylose, 25% D-galactose
8	33.3 % D-galactose, 33.3 % Maltose, 33.3 % Lactose
9	33.3 % Maltose, 66.7 % D-xylose
10	33.3 % D-glucose, 66.7 % D-galactose
11	25% D-glucose, 25% Maltose, 25 % D-xylose, 25% D-galactose
12	80% D-glucose, 20% Maltose
13	33.3 % Maltose, 66.7 % D-xylose
14	66.7 % D-glucose, 33.3 % D-xylose

Table S5. Comparison of theoretical and calculated amounts of D-glucose, Maltose, and D-xylose utilizing amperometric biosensor responses and spectrophotometric analysis.

Theoretical concentration, mM		Amperometric biosensor		Spectrophotometric analysis	
		Response, nA	Calculated concentration, mM	Absorbance	Calculated concentration, mM
D-glucose	0.025	43±3	0.026 ±0.002	0.071 ± 0.012	0.024 ± 0.004
	0.05	84 ± 5	0.052 ± 0.03	0.130 ± 0.015	0.044 ± 0.006
	0.075	126 ± 6	0.078 ± 0.004	0.206 ± 0.016	0.070 ± 0.006
	0.1	161 ± 8	0.100 ± 0.005	0.293 ± 0.01	0.100 ± 0.004
	0.15	240 ± 6	0.149 ± 0.004	0.423 ± 0.009	0.144 ± 0.003
	0.2	337 ± 5	0.209 ± 0.003	0.57 ± 0.01	0.196 ± 0.003
Maltose	0.025	32 ± 4	0.025 ± 0.003	0.142 ± 0.02	0.027 ± 0.004
	0.05	66 ± 4	0.050 ± 0.003	0.248 ± 0.012	0.047 ± 0.002
	0.075	94 ± 5	0.071 ± 0.004	0.412 ± 0.016	0.078 ± 0.003
	0.1	132 ± 2	0.100 ± 0.002	0.529 ± 0.016	0.100 ± 0.003
	0.15	191 ± 3	0.144 ± 0.002	0.797 ± 0.025	0.151 ± 0.005
	0.2	276 ± 6	0.209 ± 0.005	1.050 ± 0.02	0.199 ± 0.004
D-x	0.025	23 ± 2	0.023 ± 0.002	0.123 ± 0.016	0.028 ± 0.004

	0.05	45 ± 1	0.045 ± 0.001	0.230 ± 0.023	0.053 ± 0.005
	0.075	72 ± 5	0.072 ± 0.005	0.336 ± 0.039	0.077 ± 0.009
	0.1	100 ± 5	0.100 ± 0.005	0.436 ± 0.011	0.100 ± 0.003
	0.15	147 ± 8	0.147 ± 0.008	0.662 ± 0.028	0.152 ± 0.006
	0.2	198 ± 5	0.198 ± 0.005	0.868 ± 0.027	0.199 ± 0.006

Table S6. Concentrations of saccharides obtained by the amperometric biosensor and using spectrophotometric analysis.

Sample Nr.	Theoretical concentration, mM	Amperometric biosensor		Spectrophotometric analysis	
		Measured	Recalculated*	Measured	Recalculated*
1	0.1	0.086 ± 0.005	0.107 ± 0.005	0.140 ± 0.005	0.096 ± 0.005
2	0.1	0.088 ± 0.004	0.108 ± 0.004	0.126 ± 0.007	0.091± 0.007
3	0.1	0.091 ± 0.006	0.115 ± 0.006	0.118 ± 0.001	0.092 ± 0.001
4	0.1	0.070 ± 0.002	0.106 ± 0.002	0.155 ± 0.004	0.093 ± 0.004
5	0.12	0.115 ± 0.004	0.125 ± 0.004	0.134 ± 0.003	0.112 ± 0.003
6	0.05	0.054 ± 0.002	0.041 ± 0.002	0.061 ± 0.004	0.053 ± 0.004
7	0.08	0.079 ± 0.002	0.100 ± 0.002	0.102 ± 0.002	0.076 ± 0.002
8	0.075	0.061 ± 0.002	0.086 ± 0.002	0.142 ± 0.003	0.100 ± 0.003
9	0.1	0.075 ± 0.002	0.115 ± 0.002	0.159 ± 0.004	0.096 ± 0.004
10	0.15	0.150 ± 0.003	0.152 ± 0.003	0.134 ± 0.006	0.132 ± 0.006
11	0.04	0.031 ± 0.003	0.038 ± 0.003	0.051 ± 0.004	0.028 ± 0.004
12	0.025	0.022 ± 0.003	0.023 ± 0.003	0.030 ± 0.003	0.019 ± 0.003
13	0.025	0.023 ± 0.004	0.026 ± 0.004	0.040 ± 0.003	0.016 ± 0.003
14	0.03	0.026 ± 0.002	0.030 ± 0.002	0.034 ± 0.002	0.032 ± 0.002

*Equation S1 and S2 was used to recalculate concentration value to take into account the different specificity of the substrate or the different extinction coefficient

$$c_{analyte} = \frac{c_0}{j_0} * \sum_{i=1}^{n} \frac{j * x}{sp} + \cdots + \frac{j * x_n}{sp_n} \quad \text{(S1)}$$

$$c_{analyte} = \sum_{i=1}^{n} \frac{A * x}{\varepsilon} + \cdots + \frac{A * x_n}{\varepsilon_n} \quad \text{(S2)}$$

Here, c_0—analyte concentration used for calibration; j_0—response for c_0; sp—specificity for substrate; j—response for sample; x—part of the saccharide in the analyzed sample; A—absorbance; ε—extinction coefficient.

Table S7. Comparative performance of bioelectrode based on PQQ-GDH toward D-glucose.

Electrode	Linear range, mM	Sensitivity, μA mM^{-1} cm^{-2}	Selectivity for other saccharides	Stability	Reference
PQQ-GDH/ferricyanide/CNTs	1-35	31.0	**n/a**	87% after 28 days	[1]
GDH/PQQ@4-APPA-SWCNT-GC	0.01-0.3	13.9	**n/a**	89% after 1 day	[2]
PQQ-GDH/ Carbon paste-SPE	0.1-0.6	7.2	Nonselective	17% after 25 days	[3]
rGO_MG/PQQ-GDH	0.025-0.15	39	Nonselective	80% after 20 days	This work

Reference

1. Li, G.; Xu, H.; Huang, W.; Wang, Y.; Wu, Y.; Parajuli, R. A Pyrrole Quinoline Quinone Glucose Dehydrogenase Biosensor Based on Screen-Printed Carbon Paste Electrodes Modified by Carbon Nanotubes. *Meas Sci Technol* **2008**, *19*, 065203, doi:10.1088/0957-0233/19/6/065203.
2. Quintero-Jaime, A.F.; Conzuelo, F.; Cazorla-Amorós, D.; Morallón, E. Pyrroloquinoline Quinone-Dependent Glucose Dehydrogenase Bioelectrodes Based on One-Step Electrochemical Entrapment over Single-Wall Carbon Nanotubes. *Talanta* **2021**, *232*, 122386, doi:10.1016/j.talanta.2021.122386.
3. Razumiene, J.; Vilkanauskyte, A.; Gureviciene, V.; Barkauskas, J.; Meskys, R.; Laurinavicius, V. Direct Electron Transfer between PQQ Dependent Glucose Dehydrogenases and Carbon Electrodes: An Approach for Electrochemical Biosensors. Electrochim Acta **2006**, 51, 5150–5156, doi:10.1016/j.electacta.2006.03.058.
